# A critical role for Piezo2 channels in the mechanotransduction of mouse proprioceptive neurons

**DOI:** 10.1038/srep25923

**Published:** 2016-05-17

**Authors:** Danny Florez-Paz, Kiran Kumar Bali, Rohini Kuner, Ana Gomis

**Affiliations:** 1 Instituto de Neurociencias de Alicante, Universidad Miguel Hernández-Consejo Superior de Investigaciones Científicas, 03550 San Juan de Alicante, Spain; 2 Institute for Pharmacology, University of Heidelberg, Im Neuenheimer Feld 366, 69120 Heidelberg, Germany

## Abstract

Proprioceptors are responsible for the conscious sensation of limb position and movement, muscle tension or force, and balance. Recent evidence suggests that Piezo2 is a low threshold mechanosensory receptor in the peripheral nervous system, acting as a transducer for touch sensation and proprioception. Thus, we characterized proprioceptive neurons in the mesencephalic trigeminal nucleus that are involved in processing proprioceptive information from the face and oral cavity. This is a specific population of neurons that produce rapidly adapting mechanically-activated currents that are fully dependent on Piezo2. As such, we analyzed the deficits in balance and coordination caused by the selective deletion of the channel in proprioceptors (conditional knockout). The data clearly shows that Piezo2 fulfills a critical role in a defined homogeneous population of proprioceptor neurons that innervate the head muscles, demonstrating that this ion channel is essential for mammalian proprioceptive mechanotransduction.

Proprioceptive neurons of the peripheral sensory ganglia innervate muscle spindles, Golgi tendons and joints, playing an essential role in motor control by providing information about the body’s position, its movement and balance^1^. In a recent study, two mouse Cre lines (*Pvalb*-*Cre* and *HoxB8-Cre*) were used to target a population of sensory neurons that included proprioceptive neurons and a subset of cutaneous mechanoreceptors^2^. In these animals, mechanical stimulation of these peripheral neurons evoked non-selective, cationic, rapidly adapting (RA) currents that were dependent on Piezo2 expression, as well as intermediately adapting (IA) currents that were independent of Piezo2. Indeed, Piezo2-deficient mice develop deficits in peripheral proprioception, reflected by their abnormal movement and limb position, supporting a significant role for Piezo2 in proprioception.

In order to clearly define the role of Piezo2 in proprioception, we have characterized the mechanically activated (MA) currents in neurons of the mesencephalic trigeminal nucleus (MTN), a unique population of purely proprioceptive neurons in the brainstem^3^,^4^,^5^,^6^. The homogeneous population of neurons in this nucleus represents a much cleaner system to analyze proprioception than the peripheral sensory ganglia that contain mixed populations of sensory neurons, including proprioceptors, touch receptors, nociceptors and thermoreceptors^7^,^8^,^9^,^10^,^11^. These functionally homogeneous trigeminal mechanosensory neurons extend axons that selectively innervate the muscle spindles of the jaw-closing muscles, mediating the stretch reflex and the activity of the periodontal ligaments^12^,^13^. Using a combination of electrophysiological recordings and single cell mechano-stimulation, for the first time we show that MTN neurons develop rapidly-inactivating mechanosensitive currents. Moreover, through a genetic deletion strategy and the delivery of viral constructs, we demonstrate that these mechanically activated currents are dependent on the ion channel Piezo2. Finally, we analyzed the defects in balance and coordination caused by the selective deletion of the Piezo2 channel in proprioceptors (conditional knockout). We found that Piezo2 is essential for mechanotransduction in these central proprioceptive neurons and therefore, in proprioception *per se*.

## Results

**Figure 1:**
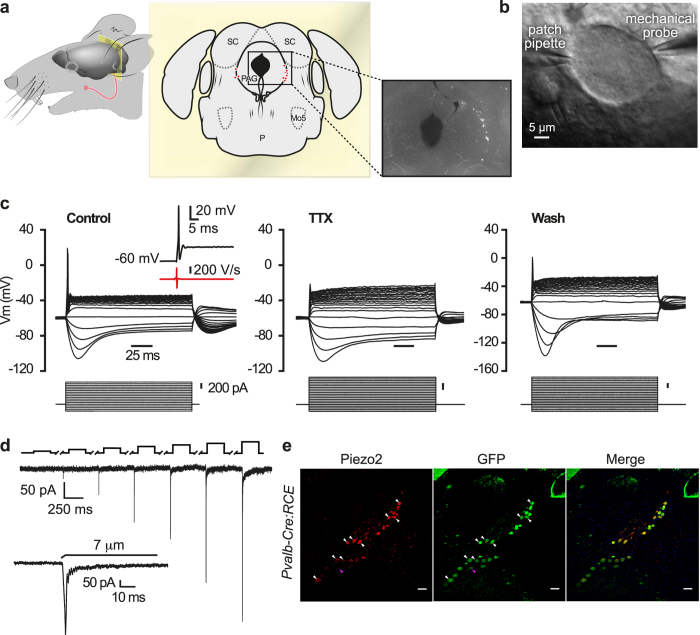
Functional characterization of MTN neurons and their MA currents in adult C57BL/6J mice. (**a**) The head of a mouse showing the muscle injection site (left). Location of the labeled MTN in a coronal section of the midbrain (middle). Fluorescent image of DiI-labeled MTN neurons in a mouse brain slice at the same location. (**b**) Phase contrast image of a MTN neuron in a brain slice showing the recording pipette and the mechanical probe. (**c**) Voltage responses (upper traces) evoked by rectangular current pulses (lower trace) in MTN neurons recorded in control conditions (n = 240), during superfusion of TTX (1μM) and after washout (n = 9). The inset shows an AP trace (black trace) evoked in MTN neurons by a depolarizing current pulse and the time derivative (red trace). The black trace represents a narrow uninflected AP whose first negative dV/dt has only one negative peak. (**d**) The RA inward current induced by mechanical indentation with the membrane potential held at −60 mV. Black upper trace shows the 250 ms mechanical stimulus steps where 1 μm increments were applied every 10 s. The breaks in the trace correspond to an equivalent break in the recording. The inset shows a higher magnification of the current trace corresponding to a 7 μm displacement. (**e**) Brainstem coronal sections from *Pvalb-Cre:RCE* mice showing MTN neurons immunolabeled for Piezo2 (n = 24; left, red) or GFP (n = 25; middle, green: parvalbumin-positive neuron) and the merged image of both (n = 24) with the nuclei stained with DAPI (blue). Representative MTN neurons are indicated by white arrowheads for neurons expressing Piezo2 and GFP, and by pink arrowheads for the GFP^+^ neuron negative for Piezo2. Scale bar 50 μm.

To identify MTN neurons in adult C57BL/6J mice, we injected a retrograde fluorescent dye (DiI) into the masseter muscle and 4–7 days later, the MTN neurons were evident as a column of labeled pseudounipolar neurons, with a large, spherical or oval soma and a mean area of 520 ± 15 μm^2^ (n = 240, Fig. 1a *left*). These neurons were located within the rostral pons and at all rostro-caudal levels of the midbrain (Fig. 1a *center* and *right*). The masseter muscle of transgenic *Pvalb-Cre* mice line was also injected with DiI, these mice expressing Cre recombinase in all proprioceptive neurons and in a fraction of RA low-threshold cutaneous mechanoreceptors^11^. These mice were crossed with the Cre-dependent EGFP reporter line (RCE:FRT)^14^ and as expected, all proprioceptive MTN neurons in these *Pvalb-Cre:RCE* mice expressed GFP. Since no cutaneous mechanoreceptors are found in the MTN, all the cells labeled with DiI also expressed GFP. We did not observe any differences in these MTN neurons and thus, in this study we pooled the data obtained from neurons labeled with DiI, GFP or with both.

MTN neurons were identified in brainstem slices under epifluorescence illumination and to define their passive membrane properties (Table 1), they were recorded by patch-clamp in the current- or voltage-clamp configuration (Fig. 1b). Injection of depolarizing current pulses evoked phasic action potential (AP) firing of virtually all MTN neurons (Fig. 1c, *left panel*), with only 2 out of 240 showing tonic discharge (data not shown). In addition, hyperpolarizing current pulses evoked inward rectification in these neurons^15^ (Fig. 1c). The MTN neurons in mice had very narrow APs with no inflection in the falling phase, as witnessed by the first derivative of the voltage (insert Fig. 1c, *left panel*). Moreover, APs were abolished by tetrodotoxin (TTX, 1 μM) but they were recovered after TTX washout, indicating that they are sustained by TTX-sensitive sodium channels (Fig. 1c, *middle and right panels*). Together these data confirm that the entire population of labeled MTN neurons belongs to the non-nociceptive, low threshold mechanoreceptor class of primary sensory neurons^16^,^17^.

To study the currents evoked by sustained mechanical indentation of the labeled neurons, a heat-polished glass pipette was applied to the soma and their responses were recorded using the voltage clamp configuration at a holding potential of −60 mV. Mechanical indentation of proprioceptive neurons exclusively provoked RA currents (Fig. 1d: mean current amplitude 144 ± 13 pA at 7 μm, n = 69; inactivation time constant τ = 4.4 ± 0.3 ms, n = 69), and no slowly adapting or intermediately adapting MA currents were detected. The amplitude of the evoked MA currents was roughly proportional to the magnitude of indentation, with a mechanical threshold of 2.9 ± 0.1 μm (Fig. 1d, n = 78: the inset shows the current trace corresponding to a 7 μm displacement at higher magnification). The reversal potential (15.4 ± 3.7 mV; n = 10) obtained from the I-V relationship of the RA currents measured after a 5 μm displacement (data not shown) indicates these are non-selective cationic currents.

**Figure 2:**
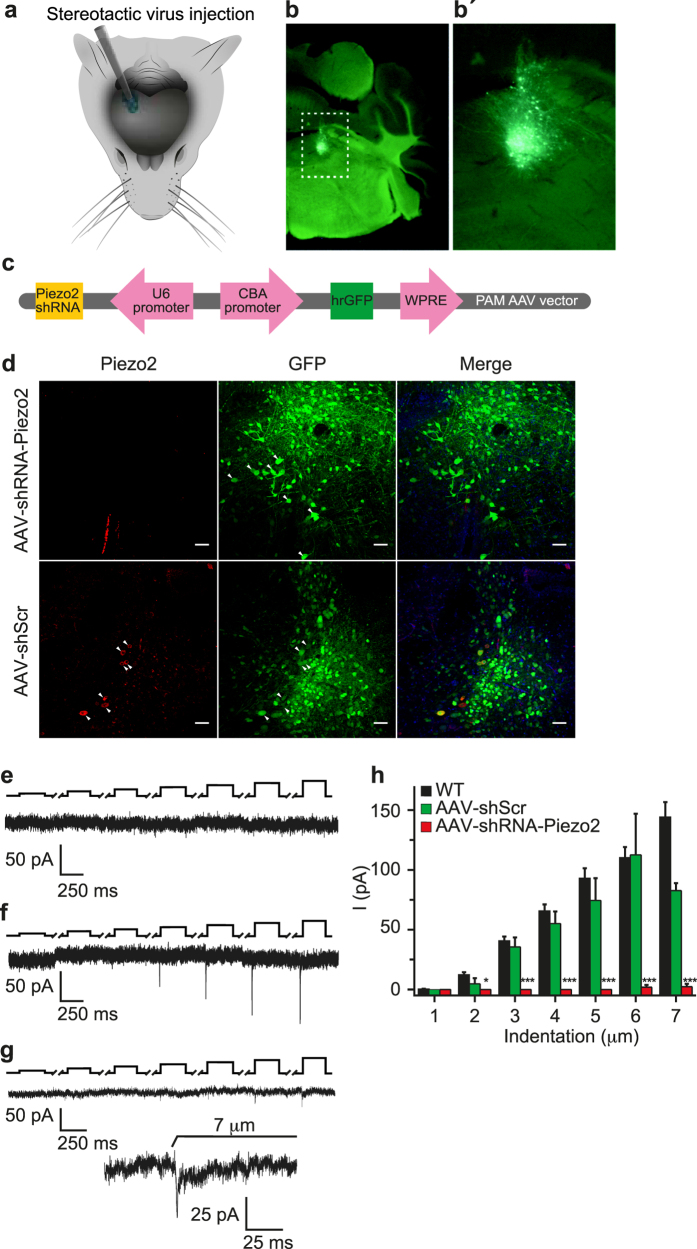
Suppression of MA in MTN neurons from adult C57BL/6J mice by silencing Piezo2. (**a**) Schematic representation of the unilateral adeno-associated viral (AAV) injection site targeting MTN neurons. (**b**) Fluorescence images showing the expression of GFP-tagged MTN neurons in brain slices following AAV delivery. (**b’**) Higher magnification of the boxed area in (**b**). (**c**) The AAV-Piezo2-sh-1 construct. (**d**) Brainstem coronal sections of C57BL/6J mice infected with the AVV-shRNA against Piezo2 (middle) and the scrambled AVV (lower), showing MTN neurons immunolabeled for Piezo2 (left, red) or GFP (middle, green), and the merged image of both, with the nuclei stained with DAPI (blue). Arrowheads indicate representative MTN neurons. Scale bar 50 μm. (**e**) Traces of MA currents evoked by mechanical indentation in MTN neurons infected with AAV-Piezo2-sh-1 or with a scrambled AVV (**f**). (**g**) Trace from a MTN neuron infected with AAV-Piezo2-sh-1 that responded with RA mechanical activated currents (τ = 6.2 ms). The inset shows a higher magnification of the current trace corresponding to a 7 μm displacement. The black upper trace shows the 250 ms mechanical stimulus steps in 1 μm increments applied every 10 s, the breaks in the trace corresponding to equivalent breaks in the recording. (h) Histogram summarizing the mean amplitude of the RA currents in MTN neurons from WT mice (n = 69) and those infected with AAV-Piezo2-sh-1 (n = 20) or AAV-shScr (n = 8). The data are expressed as the means ± s.e.m.: *p < 0.05 (Student’s *t-*test).

The mechanosensitive channel Piezo2 is a good candidate to mediate mechanotransduction in MTN neurons^18^,^19^. Indeed, when we studied the expression of Piezo2 in the *Pvalb-Cre:RCE* mouse line by immunostaining for both Piezo2 and GFP, almost every GFP^+^ neuron also expressed Piezo2 (Piezo2 expression was not detected in only 1 of the 55 GFP^+^ neurons; Fig. 1e). Thus, we examined whether the characteristic RA MA currents in MTN neurons were mediated by Piezo2, silencing its expression in MTN neurons of wild type C57B1/6J mice by infecting them with an AAV carrying a shRNA against Piezo2 (AAV-Piezo2-sh-1, Fig. 2a). We first confirmed that serotype 2/8 rAAV particles expressing GFP targeted MTN neurons (Fig. 2b,b’) and thereafter, we silenced Piezo2 using AAV-Piezo2-sh-1 (Fig. 2c). In contrast to the scrambled control shRNA (AAV-shScr) (Fig. 2d *lower*), there was a complete knockdown of Piezo2 expression after infection with AAV-Piezo2-sh-1 (Fig. 2d *top*), which was confirmed immunohistochemically. Notably, the RA MA currents were fully abolished in 19 AAV-Piezo2-sh-1 neurons (Fig. 2e,h) and in only one neuron were small amplitude RA currents evoked by indentations >6 μm (Fig. 2g,h). By contrast, the RA MA currents in neurons infected with AAV-shScr remained unaltered (Fig. 2f,h). Conversely, depolarization-induced AP firing was identical to wild type C57B1/6J mice in both types of infected animals (data not shown), and the resting membrane potential values, input resistance (R_in_) and rheobase were similar in both types of infected mice (Table 1).

**Figure 3:**
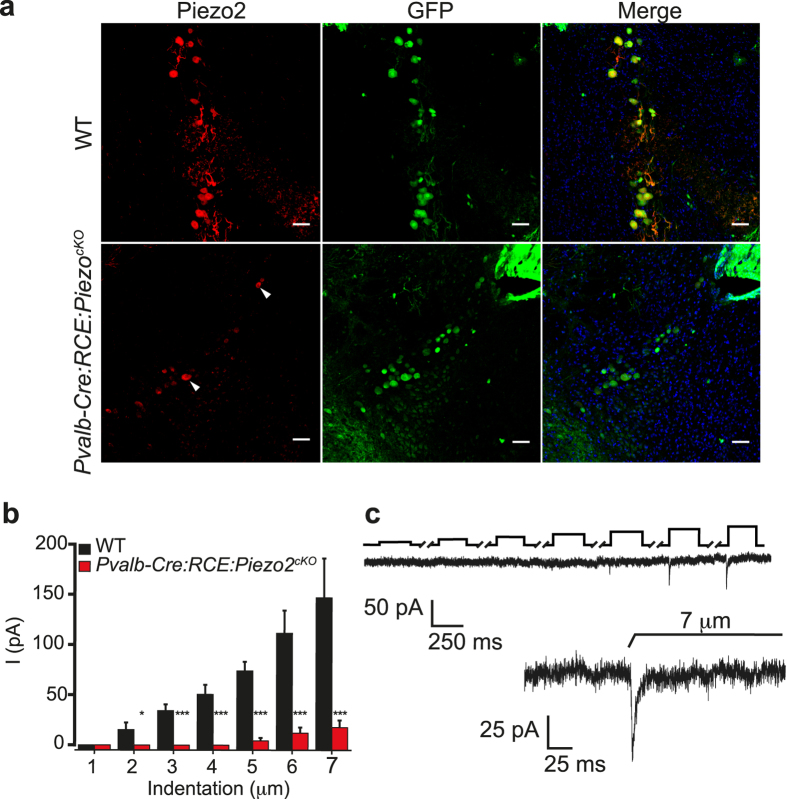
Piezo2 expression and mechanical characterization of MTN neurons in Piezo2^cKO^ mice. (**a**) Coronal brainstem sections of MTN neurons with DAPI stained nuclei (blue) that were immunolabeled for Piezo2 (red, left) or GFP (green, middle: parvalbumin-positive cell) and the merged image of both. In WT littermates mice Piezo2 was detected in the 23 GFP^+^ MTM neurons (top) and Piezo2 was detected in 7 MTM neurons of the 20 that expressed GFP in *Pvalb-Cre:RCE:Piezo2*^*cKO*^ mice (bottom; arrowhead, piezo2^+^ neuron). (**b**) Mean RA current amplitude evoked by mechanical stimuli of increasing intensity in MTN neurons of *Pvalb-Cre:RCE:Piezo2*^*cKO*^ mice (n = 15) and their WT littermates (n = 7): *p < 0.05 (Student’s *t* test). (**c**) Example of a trace from a MTN neuron of Piezo2^cko^ mice that responded with RA mechanical activated currents (τ = 4.2 ms). The black upper trace shows the 250 ms mechanical stimulus steps in 1 μm increments applied every 10 s with the breaks in the trace corresponding to equivalent breaks in the recording. The inset shows a higher magnification of the current trace corresponding to a 7 μm displacement.

To further analyze the functional role of Piezo2 in proprioception in the intact animal, we used a conditional Piezo2 knockout (Piezo2^cKO^) mouse obtained by crossing *Pvalb-Cre:RCE* with *Piezo2*^*fl/fl*^mice^20^. Immunostaining demonstrated an almost complete overlap of GFP^+^ labeling (Pvalb^+^ neurons) with Piezo2 in the MTN neurons of control littermates, only 3 of 68 GFP^+^ neurons apparently failed to express Piezo2 (Fig. 3a *top*). By contrast, 76% of the Pvalb^+^ MTN neurons in *Pvalb-Cre:RCE:Piezo2*^*cKO*^ mice do not express Piezo2 (n = 45; Fig. 3a, *lower*). The resting membrane potential of labeled MTN neurons in whole-cell recordings from the *Piezo2*^*cKO*^ mice was no different to that in their WT littermates (Table 1), nor was AP firing altered in response to depolarizing currents (data not shown). Nonetheless, the R_in_ increased and the rheobase decreased significantly when MTN neurons failed to express Piezo2 compared to the WT controls (Table 1). Mechanical stimulation did not produce a response in 10 Piezo2^cKO^ MTN neurons, while a residual RA current was provoked in the remaining 5 neurons when mechanical indentation exceeded 5 μm (Fig. 3b,c).

**Figure 4:**
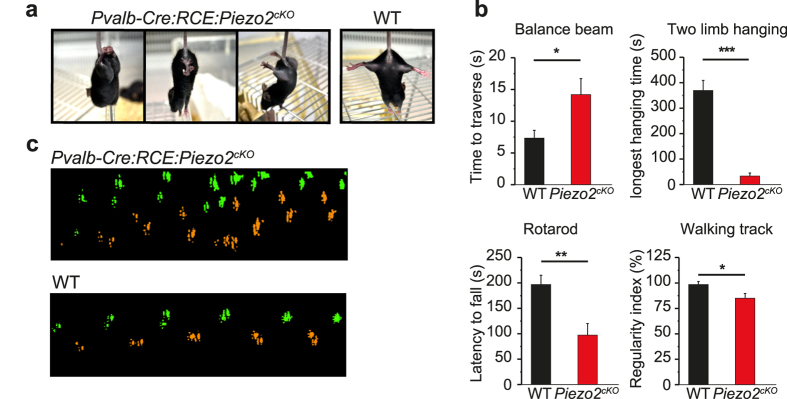
Functional characterization of Piezo2^cKO^ mice. (**a**) Images of 6–9 week-old Piezo2^cKO^ mice showing clear hindlimb retraction and clasping (left and middle), and widely splayed hindlimbs when lifted by their tails. None of these postures were observed in their WT control littermates (right). (**b**) Behavior of *Pvalb-Cre:RCE:Piezo2*^*cKO*^ mice (n = 7) and their WT littermates (n = 7) in balance and coordination tests. (**c**) Step sequence of *Pvalb-Cre:RCE:Piezo2*^*cKO*^ mice and their control littermates, showing the order in which the paws were placed when the animal crossed a walkway with an illuminated glass floor (as tested with the Catwalk system); right fore and hind paws in green, and left fore and hind paws in orange. The data are expressed as the mean ± s.e.m.: *p < 0.05 (Student’s *t* test; Mann-Whitney Rank Sum).

The position of the limbs was abnormal in Piezo2^cKO^ mice (Fig. 4a) and 6–9 week old mice of either sex had a lower body weight than their control littermates (data not shown), perhaps reflecting a malfunction of the muscles driving mastication and of the periodontal pressoreceptors. We analyzed the disturbance in balance, muscle function, limb mobility and coordination in these animals using the balance beam, two limb hanging, and the rotarod and catwalk tests, respectively. Compared to their control littermates, Piezo2^cKO^ required three times as long to traverse the beam, while their latency to fall off a metal wire upon exhaustion or their walking time was only few seconds. In addition, their degree of limb coordination was clearly diminished (Fig. 4b,c; Movies 1–3).

## Discussion

Our results define MTN neurons as a unique, homogenous population of proprioceptive neurons that have morphological and functional properties typically associated to low threshold mechanoreceptors: a large soma, narrow AP and sensitivity to TTX. To our knowledge, this is the first formal characterization of the mechanical properties of MTN neurons in which mechanical forces exclusively evoke RA non-selective cationic currents. Unlike DRG recordings from *Pvalb-Cre:Piezo2*^*cKO*^ mice (Woo *et al*.^2^), we did not record IA currents in MTN neurons, which suggests that the IA currents displayed in the DRG of these animals could be generated by the cutaneous mechanoreceptors that are also expressed by parvalbumin + DRG neurons. Moreover, the RA currents mechanically activated in MTN neurons are linked to the expression of Piezo2. Indeed, selective deletion of this ion channel in MTN neurons through AAV or genetic deletion strategies almost completely abolished their RA mechanically-evoked currents. Furthermore, the lack of Piezo2 in proprioceptive neurons severely disturbed balance and coordination, evidence that this transducer channel is critical in conferring mechanical sensitivity generally attributed to proprioceptive sensory neurons.

Viral vector infection was more effective in silencing Piezo2 than genetic ablation, as witnessed by the virtually complete depletion of MA currents in AAV infected MTN neurons. Indeed, Piezo2 was not detected in these cells by immunostaining. Nevertheless, small amplitude MA currents could be recorded in both types of Piezo2 deficient cells, although the mechanical force required to activate these currents was higher than in the control cells. This could reflect the incomplete silencing of Piezo2 in those neurons, in which it is necessary to apply more intense stimulation in order to recruit and activate the remaining Piezo2 channels to evoke a response. However, it is possible that the cells that maintain some mechanosensitivity in the absence of Piezo2 express other mechanosensitive channels that are activated by stronger mechanical forces and that contribute to the proprioceptive sensations of effort, force and heaviness^1^,^21^.

In MTN neurons from Piezo2^ckO^ mice, the rheobase decreased and the input resistance increased, indicative of a lower discharge threshold as also seen in DRG neurons^2^. As the resting potential did not change, such differences can be explained by the compensatory gain-of-function of other channels that enhance the excitability of Piezo2 deficient neurons. However, this would not explain the changes in rheobase and input resistance in animals infected with the control AVV-shRNA-scrambled virus. The decrease in rheobase and the increase in input resistance was similar in both types of virus treated mice but statistically different from the values in non-infected mice (WT mice in Table 1), suggesting that the AAV virus itself might be responsible for these changes. Finally, mice lacking Piezo2 in proprioceptive neurons display severe alterations to limb position and impaired performance in different behavioral tests, indicative of neurological deficits.

This study clearly shows a critical role for Piezo2 in a homogeneous population of proprioceptor neurons that innervate the muscles of the head. Together with a recent study characterizing proprioceptors in DRG neurons^2^, these data convincingly identify the transducer of proprioception. The MTN contains the cell bodies of primary afferent neurons with proprioceptive functions related to the teeth and mastication. These neurons project directly to motor neurons of the trigeminal motor nucleus, providing a rapid monosynaptic reflex. Considering that the MTN is a crucial component of the neural circuitry responsible for generating and controlling oromotor activities, these data advance our understanding of the mechanisms and networks underlying motor control. Finally, our results may provide new insights into pathological aspects of proprioception, which might in turn lead to a more objective assessment of its clinical defects.

## Materials and Methods

### Animals

All experimental procedures were performed according to the Spanish Royal Decree 1201/2005 and the European Community Council directive 2010/63/EU regarding the handling of experimental animals. This study was approved by the Ethics Committee at the Universidad Miguel Hernández, Alicante, Spain.

### Mouse Lines

Adult C57Bl/6J mice were used in this study. We generated the *Pvalb-Cre:RCE:Piezo2^fl/fl^ cKO* mice on this background by crossing *Pvalb-Cre*^22^ with floxed *Piezo2*^20^ mice, expressing a Rosa26 reporter CAG-boosted EGFP^14^. Male animals were used for *in vitro* recordings and either sex was used for the *in vivo* experiments.

### Dye injections

To label MTN neurons, animals were anesthetized by isoflurane inhalation, and the lateral surfaces of their head were shaved and cleaned before making a 5 mm incision transverse to the mouth line. The masseter muscle was exposed and the DiI tracer (5%: Life Technology, Carlbad, USA) was injected bilaterally into the muscle. The incision was closed with a drop of cyanoacrylate and the DiI was allowed to diffuse retrogradely into the cell bodies in the MTN.

### Slice preparation

Coronal slices from adult mice were prepared 4–7 days after tracer injections as described previously^23^. The animals were sacrificed by cervical dislocation, decapitated, and their brain was removed rapidly and immersed in ice-cold, oxygenated (95% O_2_–5% CO_2_) artificial cerebrospinal fluid (ACSF) composed of (in mM): 120 NaCl, 2.5 KCl, 1 NaH_2_PO_4_, 26 NaHCO_3_, 2.5 CaCl_2_, 1.2 MgCl_2_ and 11 glucose. The brainstem was attached at its rostral end to the platform of the vibrating slicer (Pelco, TPI, series 1000, St. Louis, USA) and covered with ice-cold ACSF. Slices (200 μm thick, −4.16 to −5.80 mm posterior to bregma)^24^ were obtained in ACSF at 4 °C and then maintained at 34 °C.

### Whole cell recordings

Patch clamp recordings were performed in the whole cell configuration using a Multiclamp 700 amplifier, and the data were acquired using pCLAMP 10 software and a Digidata 1440 (Molecular Devices, Sunnyvale, USA), sampled at a frequency of 20 KHz and filtered at 10 KHz. Patch electrodes of 4–7 MΩ were pulled from borosilicate glass capillaries and 70% series resistance compensation was used. The pipettes were filled with an internal solution containing (in mM): 115 K-gluconate, 25 KCl, 9 NaCl, 10 HEPES, 0.2 EGTA, 1 MgCl_2_, 3 K_2_-ATP and 1 Na-GTP, pH 7.2 adjusted with KOH and 280–290 mosmol Kg^−1^. The control external solution consisted of (in mM): 140 NaCl, 3 KCl, 1 CaCl_2_ 2 MgCl_2_, 10 glucose and 10 HEPES (pH 7.2). Slices were transferred to a recording chamber and perfused with oxygenated external control solution. MTN neurons were identified under a fluorescence microscopy (Olympus BX50WI) coupled to an imaging system that included polychrome V, an Imago camera (both Till photonics GmbH, Germany) and the Imaging Workbench 6.0 software (INDEC BioSystems). As most sensory neurons are oval, their two diameters were measured and the area was derived from the ellipse equation (π* r1 * r2). Current injection was used to evoke action potentials (APs) with pulses varying from −200 pA to 1 nA over 250 ms. The following electrophysiology parameters were measured: resting membrane potential (Vm), input resistance (R_in_), rheobase, and hump or inflection in the repolarizing phase of the AP (dV/dt)^25^. Mechanically activated currents were recorded at a holding potential of −60 mV. To measure the current-voltage (I–V) relationship of the RA MA currents, the mechanical stimulus was delivered with the membrane command voltage set to different membrane potentials between −80 mV and +40 mV. The mean reversal potential was calculated as the mean of the reversal potential of each cell. All experiments were carried out at 34 °C.

### Mechanical stimulation

Mechanical stimulation of cell bodies was performed as described previously^26^,^27^, using a heat-closed glass pipette driven by a MM3A-LS piezoelectric micromanipulator (Kleindiek, Reutlingen, Germany). The mechanical pipette was positioned on a cell body at a 45–60° angle to the horizontal plane and opposite the recording pipette. The probe was moved at 3.5 μm ms^−1^ and the stimulus was applied for 250 ms. A series of mechanical steps in 1 μm increments were applied every 10 s. The RA MA currents were classified by their inactivation kinetics that were fitted with a mono exponential equation and with an inactivation time constant (τ) < 10 ms.

### Virus production and *in vivo* injection

A set of 4 shRNAs were designed against the Piezo2 coding region (Cat. No. TR509519: Origene, USA), and each shRNA sequence was cloned by standard methods into the adeno-associated viral (AAV) backbone of the PAM vector along with the U6 promoter. The expression of the native GFP reporter cassette in the final clones was confirmed by the strong GFP signal following transfection into HEK-293 cells. The rAAV serotype 2/8 particles carrying shRNA against Piezo2 (AAV-Piezo2-sh-1) or a scrambled shRNA were generated in house by co-transfecting HEK-293 cells with the cloned rAAV backbone plasmid and the helper 2/8 plasmids following standard protocols^28^. Only the 1^st^ shRNA against Piezo2 was used in this study (NCBI Reference Sequence: NM_001039485.4, gene ID: 667742; the sequence in bold represents the target binding site): 5′**TTCTGGCTGACACTGTAGACTTCATTAT**CTCAAGAGGATAATGAAGTCTACAGTGTCAGCCAGAATTTTTT-′3. Sequence of scrambled shRNA:

5′**GCACTACCAGAGCTAACTCAGATAGTACT**TCAAGAGAGTACTATCTGAGTTAGC TCTGGTAGTGCTTTTTT-′3.

Mice anaesthetized under isoflurane (2.5%) underwent surgical procedures in which the area of the skull over the midbrain was thinned and a ~2 mm^2^ craniotomy was performed. The virus was delivered using a glass micropipette controlled by a nanoliter injector microprocessor (Nanoliter2010, WPI). The needle was held for 1 minute prior to the injection and the viral solution was injected in a total volume of 700 nl and at a rate of 46 nl/s. Injections were performed at the location 5.4 mm anteroposterior to Bregma, ±1.2 mm mediolateral, 2.2 mm dorsoventral. After the full volume of virus was injected, the needle was held in place for a further minute prior to withdrawal and a layer of adhesive cement (bonewax) was applied around the hole. We then sutured the skin with a tissue adhesive (cyanocrylate). The mice were given 0.1 mg/Kg Buprenorphine as an analgesic and they remained on a heated pad until they recovered from the anesthesia. The mice were studied in the two weeks following surgery.

### Immunohistochemistry

Mice were anesthetized with ketamine (200 mg/ml) and xylacine (50 mg/ml), and they were perfused with 4% paraformaldehyde (PFA) in 0.1 M phosphate buffer (PB). The brain was then removed from each mouse and after post-fixing by immersion in 4% PFA (24 h, 4 °C), coronal vibratome sections (50 μm; VT 1000S, Leica) were obtained and then incubated for 30 min in blocking solution at room temperature (3% goat serum in TTBS). The sections were immunostained overnight at 4 °C with the primary antibodies and after washing three times in PBS (5 min) the secondary antibodies were applied for 2 h at room temperature. After three 5 min rinses with PBS, the nuclei were stained with DAPI (2 μM; Life Technology, Carlsbad, USA). The antibodies used were: rabbit anti-Piezo2 (1:100; NBP1-78624, Novus Biological, Littleton, USA); chicken anti-GFP (1:1000; AB13970, Abcam, Cambridge, UK), goat-anti rabbit AlexaFluor-594 (1:1000; A11012) and goat-anti chicken AlexaFluor-488 (1:2000; A11039, Life Technology).

### Behavioral tests

In this study we tested a cohort of 6–9 week-old female *Pvalb-Cre:RCE:Piezo2*^*cKO*^ mice (n = 7) and their control littermates (n = 7). The animals were housed on a 12 h light/dark cycle at 21 °C with food and water available *ad libitum*. Balance and coordination was examined using four tests: the balance beam, two limb hanging, rotarod, and walking track^29^,^30^,^31^. Balance was tested on a 1 m long, 2 cm wide beam suspended on two poles 50 cm above a table top. Food was placed in a box at the finish point to attract the mouse and a lamp above the start point served as an aversive stimulus. A video camera on a tripod was used to record the time required to cross the beam. The balance beam is a useful measure of fine coordination and balance.

For two limb hanging we used a 2 mm diameter metal bar mounted between two ring stands 37 cm above a cotton pad that was situated under the wire in between the stands. The mouse is brought close to the wire so that it can grasp the wire with its forelimbs, and the time is set to zero as soon as it is released and is hanging onto the wire. The latency to fall was measured and the maximum time allowed was 500 seconds.

In the rotarod test (Ugo Basile), the mice were placed on a 5 cm diameter rotating cylinder along which they must walk in order not to fall off. The initial speed of rotation was 5 rpm and it accelerated to 20 rpm. The animal’s gait was tested with the CatWalk system 7.1 (Noldus Information Technology), where the mice were placed in a glass chamber (85 cm long and 8.5 cm wide) and they were allowed to move freely across the walkway. Fluorescent light is emitted inside the apparatus and reflected internally. The contact of the paws with the glass reflects the light, allowing the paw prints to be captured by a video camera and analyzed with the CatWalk software. We analyzed the regularity index (%) that expresses the number of normal step sequence patterns relative to the total number of paw placements, a fractional measure of inter-paw coordination. In healthy, fully coordinated animals this value is 100%.

The same investigator scored all the tests, each of which was repeated at least three times in each animal, and the values from the individual tracks were averaged. All the animals were habituated and trained in the different tests prior to performing the experiment.

### Statistical analysis of the data

The data were analyzed with the Clampex 10.1 software (Molecular Devices Corp., Sunnyvale, CA, USA) and Origin 7.5 (Origin Lab Corporation, Northampton, MA, USA). Statistical analyses were performed with SigmaStat (Systat Software Inc.) and the data are reported as the mean ± SEM: *P < 0.05, **P < 0.01, ***P < 0.001 as assessed by the Student’s *t*-test and Mann-Whitney Rank Sum test.

## Additional Information

**How to cite this article**: Florez-Paz, D. *et al*. A critical role for Piezo2 channels in the mechanotransduction of mouse proprioceptive neurons. *Sci. Rep.* **6**, 25923; doi: 10.1038/srep25923 (2016).

## Supplementary Material

Supplementary Information

Supplementary Information

Supplementary Information

Supplementary Information

## Figures and Tables

**Table 1:** Electrical properties of MTN neurons from *Pvalb-Cre:RCE* (WT) and *Pvalb-Cre:RCE:Piezo2*^*cKO*^ mice, and of MTN neurons infected with AAV-Piezo2-sh-1 or with a scrambled AAV.

	**WT (n = 240)**	**Piezo2**^**cKO**^ **(n = 15)**	**Piezo2**^**cKO**^ **(wt littermates) (n = 7)**	**AAV- Piezo2-sh-1 (n = 20)**	**Scrambled (n = 8)**
Resting Membrane potential (mV)	−51 ± 0.7	−49 ± 2	−50.3 ± 2.1	−49 ± 1.2	−51 ± 2.6
Input resistance (MΩ)	125.3 ± 5.2	154.7 ± 15.6 (**)	76 ± 22	209.5 ± 41.7 (###)	180 ± 33 (###)
Rheobase current (pA)	620 ± 16	363 ± 39 (*)	685 ± 116	322 ± 38 (###)	319 ± 60 (###)

Comparisons were made between Piezo2^cKO^ and their WT littermates (*), MTN neurons infected with AAV-Piezo2-sh-1 or with a scrambled AAV and MTN neurons infected with AAV-Piezo2-sh-1 or a scrambled AAV with neurons from WT mice (^#^): *,^#^ denotes p < 0.05; Student *t*-test and Mann-Whitney Rank Sum test.
