# Supplementary material for: A critical role for Piezo2 channels in the mechanotransduction of mouse proprioceptive neurons
Source: Sci Rep. 2016 May 17;6:25923. doi: 10.1038/srep25923 (PMC4869095; doi:10.1038/srep25923)
Supplement: Supplementary Information [file srep25923-s4.pdf]

Supplemementary Information

A critical role for Piezo2 channels in the Mechanotransduction of Mouse Proprioceptive Neurons

Danny Florez-Paz, Kiran Kumar Bali, Rohini Kuner and Ana Gomis

Video legends

Video 1: balance beam test performed in WT and Piezo^{cKO} mice.

Video 2: two limb hanging test performed in WT and Piezo^{cKO} mice.

Video 3: rotarod test performed in WT and Piezo^{cKO} mice.
